# Immediate effect of ice and dry massage during rest breaks on recovery in MMA fighters : a randomized crossover clinical trial study

**DOI:** 10.1038/s41598-025-97194-x

**Published:** 2025-04-10

**Authors:** Robert Trybulski, Arkadiusz Stanula, Andriy Vovkanych, Jarosław Muracki, Hsing-Kuo Wang, Adrian Kużdżał

**Affiliations:** 1 Medical Department, Wojciech Korfanty Upper Silesian Academy, Katowice, 40-659 Poland; 2 Department of Physical Therapy and Ergotherapy, Ivan Boberkyj Lviv State University of Physical Culture, Lviv, 79007 Ukraine; 3 Laboratory of Sport Performance Analysis, Institute of Sport Sciences, Jerzy Kukuczka Academy of Physical Education in Katowice, Katowice, 40-065 Poland; 4 Institute of Physical Culture Sciences, Department of Physical Culture and Health, University of Szczecin, Szczecin, 70-453 Poland; 5 Provita Medical Center, Żory, 44-240 Poland; 6 School and Graduate Institute of Physical Therapy, National Taiwan University, Taipei, Taiwan; 7 Center of Physical Therapy, National Taiwan University, Taipei, Taiwan; 8 Institute of Health Sciences, College of Medical Sciences, University of Rzeszów, Rzeszów, 35-310 Poland

**Keywords:** Cooling, Mechanotransduction, Regeneration, Combat sport, Myotonometry, Musculoskeletal system, Rehabilitation, Pain management, Rehabilitation

## Abstract

The MMA fight consists of 5 rounds of 5 min with minimal breaks between the rounds. The exertion load is excessive for the fighters, and the 1-minute breaks give little time for any intervention. This study aimed to examine the acute effects of two methods of regenerative strategies, ice massage and dry massage, and analyze their impact on Reactive Strength Index (RSI - m s^− 1^), muscles’ biomechanical properties: muscle tone (T-Hz), elasticity (E - arb- relative arbitrary unit), stiffness (S - N/m), pressure pain threshold, (PPT - N/cm²), and compare their influence with passive rest. The maximum number of jumps (J - n) treated as an indirect effective measure of the interventions that were conducted was also recorded for each participant in each regenerative strategy. Thirty male MMA fighters took part in the study. Three subgroups of 10 participants (Ice massage, *n* = 10; dry massage, *n* = 10; and control, *n* = 10) were enrolled in the cross-over randomized clinical trial study design. The groups were randomized, and each group underwent each procedure (30 tested in each procedure). Five sets of jumps on a 50 cm box to exhaustion were used as a fatigue protocol with 1-minute breaks. The recovery interventions were performed during the breaks. The statistically significant results revealed in the post-exercise tests: RSI and number of jumps - the lowest decrease was observed in the massage group (*p* < 0.001 and *p* < 0.0001 respectively), the minor increases in T, E and S were also observed in the massage group ((*p* < 0.0001 for all measurements); the post-exercise PPT was the highest (higher means better) in the Ice group *(p* < 0.001). In every other parameter, the ice massage group showed slightly worse results than the dry massage group. Responder analysis confirms that the number of jumps profoundly impacted biomechanical variables, leading to increased muscle stiffness and tension, decreased elasticity and force endurance, and heightened pain sensitivity. Obtained results confirm that both dry and ice massage can significantly affect acute recovery following rounds of combat sport-related exertions. The Ice and Massage interventions differed in effectiveness - Massage was the most effective in preventing increases in stiffness and tension and preserving muscle elasticity. At the same time, ice cooling had a lesser impact, particularly on muscle elasticity changes but higher for PPT.

## Introduction

### Background

Mixed martial arts (MMA) requires good motor preparation because the athlete uses punching, kicking, grabbing, choking, and throwing techniques from other combat sports^1^. The scientific literature on MMA describes that mixed physical exercise predominates (aerobic and anaerobic)^2^. Sports training must include balanced aerobic and anaerobic efficiency development concerning the upper and lower limbs^3^. A professional fight consists of 3 or 5 rounds of 5 min each, while amateur fights consist of 3 rounds of 3 min each^4^. Between rounds, coaches have time to provide information regarding fighting tactics and implement quick strategies to compensate for the effects of muscle loads^5^. Eccentric contractions of the thigh muscles are a primary type of muscular effort in MMA. Eccentric training emphasizes muscle lengthening under load and has significantly improved eccentric and concentric strength and jumping performance^6^. Therefore, this type of muscular work is used in training for motor development and testing among competitors^1^.

### Influence of exertions on muscle Biomechanical properties

Research confirms that intensive exertions influence muscle biomechanical parameters. Muscle stiffness, elasticity, and muscle tone increase, while reactive strength index (RSI) - a measure that describes the individual’s capability to quickly change from an eccentric muscular contraction to a concentric one decreases after exercise^7,8^. Pressure pain threshold (PPT)—a measure defined as the minimum force applied that induces pain—increases immediately after exercise and has a prolonged effect as a reflection of muscle fibers’ damage, leading to DOMS^9^. MMA athletes are exposed to these effects as a consequence of both training sessions and competition fights. Considering that the MMA training routine contains many sessions per week, the training process needs to be accompanied by effective recovery strategies. During fights, the recovery interventions need to have an immediate effect in the low time between the rounds, which is very demanding and may be crucial for an athlete’s performance^10^.

### Recovery strategies

Periodization of MMA training reveals several factors that influence energy levels and muscle endurance in MMA, including regeneration^11^. Although no consistent protocols exist for these methods, they are widely used and practical. Popular methods include cold stimuli^12^. in the form of water immersion^13^, ice packs^14^, contrast therapy^15^, dry massage^16,17^, lymphatic drainage^18^, instrumental massage^19^, and many others^20^. These methods are not only theoretical but have practical applications in reducing DOMS^14,21^. , improving muscle load^22^, improving muscle blood supply^23^, eliminating inflammation^24^ and reducing muscle tension and stiffness^25^, thus affecting regeneration and performance^18^.

Previous research has shown active post-exercise recovery strategies are more effective than passive rest^26^. Some studies suggested no difference in performance when the recovery duration is above 15 min^26^. Trainers often use ice massage^27,]^ which is one of the most tired muscle groups, or dry massage^28^, which involves rubbing, shaking, and stroking the muscles between exertions^29^. There is no consensus in the scientific literature on the effects of massage on motor skills and athletic performance^30^. Still, evidence supports improved muscle function after massage therapy^21,31^. Several studies have been presented on muscle fatigue, likely related to creatine kinase enzyme efflux and psychological applications^32^. Massage has been shown to reduce stress and anxiety, improve post-exercise recovery^17^, and reduce inflammatory cytokines such as TNF-α and IL-6, which are elevated following muscle damage^33^. Studies suggest that massage can prevent significant increases in CK and LDH levels following exercise, suggesting a protective effect on muscle integrity^34^. Although massage benefits muscle recovery, some studies suggest its effects may vary based on individual responses and specific techniques^35^. Additionally, some studies have reported inconsistent results, failing to confirm an effect on muscle recovery^36,37^. Further research is needed to optimize massage protocols for different populations and exercise types.

Ice massage, along with sports massage, is the most common form of action in muscle regeneration after physical exercise^38^. Its popularity is primarily due to its easy access, the possibility of self-massage by the athlete, and its confirmed effectiveness in reducing muscle pain^27^. The effect of ice massage on muscle recovery has been a topic of debate, with mixed results from various studies^39^. While some studies suggest that icing may facilitate muscle recovery by modulating inflammatory responses^40^, other studies suggest it may impede recovery^41^Additionally, evidence-based recommendations are still missing on the questions of stimulation time, number of sets, and other parameters of ice massage protocols for recovery between exercises^36^.

### Aim of the study

The scientific literature describes the regenerative effects of various methods of post-MMA training^8,10,20,23,42–45^. Therefore, recognizing the gaps in knowledge described above, our study aimed to compare the recovery effectiveness of ice massage and dry massage interventions during 1 min inter-exercise breaks of eccentric exercises on the quadriceps femoris muscles in MMA athletes. The impact on muscles’ biomechanical parameters: tension, stiffness and elasticity; pressure pain threshold PPT, reactive strength index RSI and muscle power and endurance as a number of box jumps up till exhaustion was assessed. The ice massage and dry massage methods are commonly applied between the rounds, so the most practical aspects of the testing and recovery procedures are adopted.

We hypothesized that evaluating both intervention sessions would yield improved outcomes compared to a control session based on passive rest – understood as reducing muscle stiffness, tension, and elasticity, preventing lowering of the PPT and RSI, and helping to achieve more jumps compared to the control group. Still, the study’s main aim was to determine if there are significant differences between these strategies. In particular parameters, we only hypothesized that the ice massage would better prevent the PPT from decreasing and that dry massage would be better for reducing stiffness. Assessment of musculoskeletal tissue and its properties before and after applying various stimuli during breaks between exercises may be crucial in building immediate recovery strategies to facilitate athletes and continue physical exercise optimally. In addition, short-term recovery may reduce the risk of overload injuries associated with repeated exposure of muscles to high-power physical activity. The study’s results may provide general information necessary to develop practical recovery strategies in combat sports.

## Materials and methods

### Study design

**Fig. 1:**
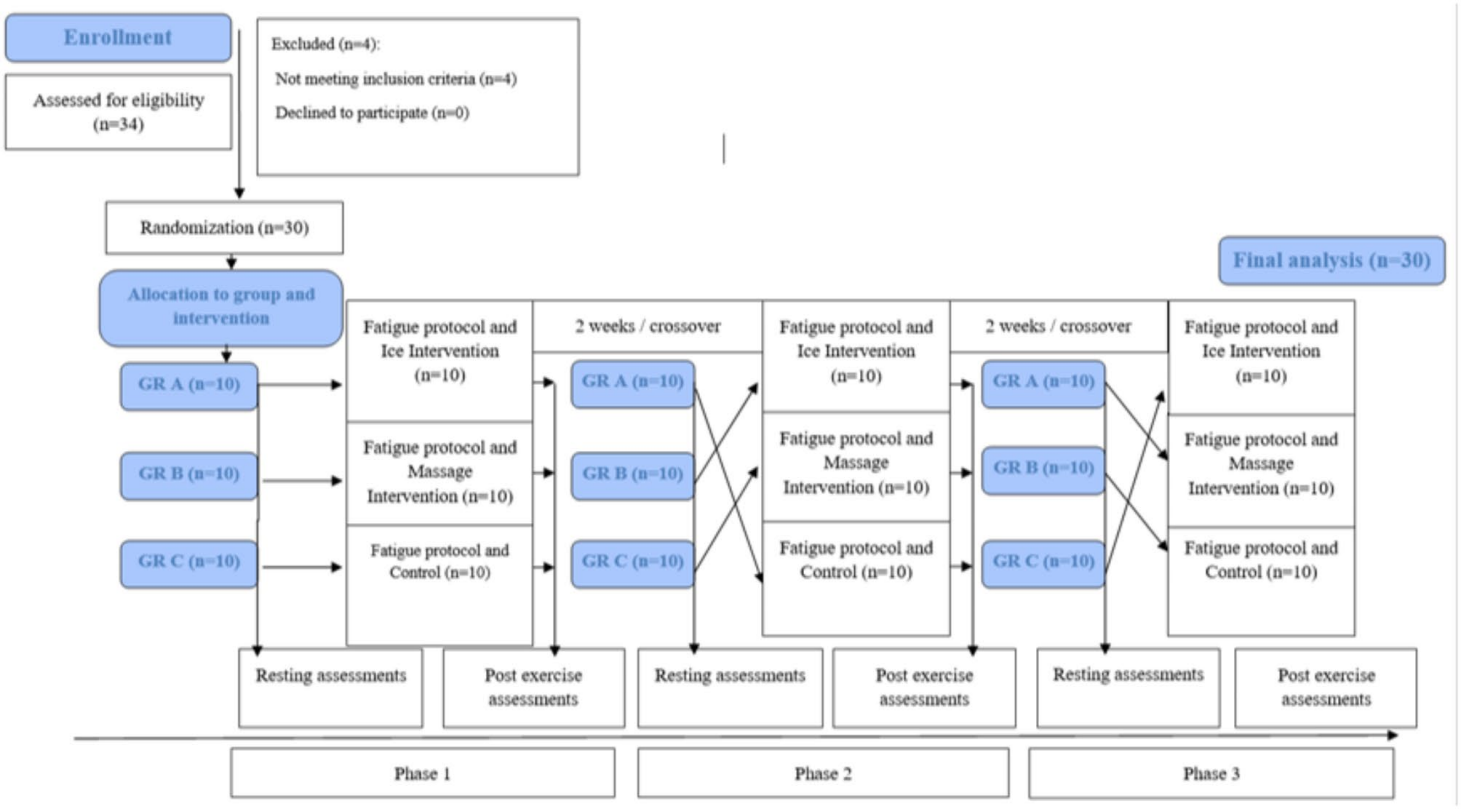
Study design.

This randomized, single-masked, crossover clinical trial included 30 MMA training volunteers (*n* = 30). The participants were recruited between 20/07/2023 and 1/08/2023, and the study was conducted in Provita Medical Center (Żory, Poland). The participants were recruited and randomly divided into three groups of ten people (A, *n* = 10, B, *n* = 10, C, *n* = 10) with different recovery interventions by the project administrator. The study design included 3 phases; in each phase, groups underwent three different treatment strategies, alternating every 2 weeks. All study participants were examined between 9:00 a.m. and 12:00 p.m. at the Provita Medical Center in Żory, Poland. Before starting the study, volunteers completed a consent form and a health questionnaire. The same conditions prevailed during the tests: air temperature 21 °C and air humidity 50–55%). Group allocation was performed by the project administrator by simple 1:1 randomization with a randomized sequence using the website randomizer.org. The group assignment was independent of treatment duration and study personnel. In addition, the order of interventions for each group was also randomized by the study administrator (Fig. 1). Each participant underwent a familiarization intervention, performing two sets of 30-second plyometric jumps with massage and ice cooling of the muscles in breaks between exercises seven days before the study. The study was approved by the ethical committee of the Polish National Council of Physiotherapists (no 24/04/2023) and registered in the ISRCTN register of clinical trials (10.1186/ISRCTN10033645, registration date: 04/07/2023), and carried out by the Declaration of Helsinki.

### Participants

The study included martial arts athletes (*n* = 30) (age: 27.2 ± 4.36 (years), BMI: 25.3 ± 3.07(kg×m^− 2^), training experience: 10.8 ± 4.76 (years) (Table 1). They met the following inclusion criteria: (i) age 18–40 years; (ii) at least 3 years of experience in combat sports training; (iii) training at least five times per week. Considering McKay’s participant classification scheme, the study participants were at tier 3: highly trained/national level^46^ .The training routine included a motor training session at least once per week, during which elements of plyometric training occurred. The exclusion criteria were as follows: (i) elevated blood pressure before the study (blood pressure > 140/90 mm Hg); (ii) currently treated injuries, damaged skin, or unspecified skin lesions at the measurement sites; (iii) tattoo at the measurement site (as this interfered with tissue perfusion measurements); (iv) taking any medication, including painkillers and hormonal agents. Athletes were also excluded in case of extreme fatigue, fever, infection, or at the explicit request of the participant at any time during the study. Written informed consent was obtained from all the participants after they were informed of the study’s conditions, advantages, and risks. Participants were required to refrain from training for 24 h before and 48 h during the study. In addition, due to tissue perfusion measurements, participants were asked to refrain from consuming any ergogenic drinks (a list of excluded products was provided to participants) 24 h before the study. The statistical summary of the group characteristic is presented in Table 1.

**Table 1:** Characteristics of the study group (*n* = 30). Data are presented as mean ± standard deviation (± 95 confidence intervals of mean) and range (minimum and maximum values).

Variables	mean ± SD	± 95 CI	Range
Age (year)	27.2 ± 4.36	25.57, 28.83	20–36
Stature (cm)	180.0 ± 7.72	177.15, 182.92	156–197
Body mass (kg)	81.0 ± 10.17	77.19, 84.79	56.3–98.8
Training experience (year)	10.8 ± 4.76	9.05, 12.61	2–20
BMI (kg×m^− 2^)	25.3 ± 3.07	24.13, 26.43	21.1–32.9

### Muscle fatigue protocol

The study used plyometric jumps known to the participants on a 50 cm high box^8^. Before the study, MMA fighters declared the ability to perform this exercise and confirmed its regular use in training (at least once a week). The protocol consisted of 5 plyometric jumps until the participant could not continue the effort. There was a 1-minute break between subsequent sets (partial conditions reflecting an MMA fight) in which the recovery intervention was conducted. Plyometric exercises are often used in combat sports training; they are eccentric motions that reduce high load and high intensity and damage the muscle cells, making it a significant load to the participants. What is more – the MMA fight induces very high fatigue – which is why we decided to use a version in which the set ends when the participant cannot continue the effort. Five sets and 1-minute breaks between the sets should replicate the actual MMA fight time structure. The initial measurements were performed before the fatigue protocol after the warm-up. Before the fatigue task, participants performed a warm-up consisting of a 5-minute ride on a bicycle ergometer at moderate intensity and a 3-minute stretching program for the leg muscles (including knee flexors and extensors, hip abductors and adductors, and triceps calves). Throughout the fatigue protocol, the participants were supervised by a paramedic and an assistant who counted and controlled the quality of the jumps performed. Many researchers recommend using box jumps in MMA training mainly because they allow for reducing ground reaction forces during landing, thus limiting the increased risk of injury often associated with excessive eccentric loads^8,29^. The box jumps consisted of the following phases: (1) Initial phase: position in front of the plyometric box, lowering the body by flexing the hips and knees and extending the ankles and arms); (2) Jump phase (concentric), dynamic jump up and forward (extending the hips, knees, and flexing the ankles and arms); (3) Load reaction phase on the box (eccentric);4. The initial phase of the jump; 5. Dynamic jump; 6. Load reaction phase on the ground^8^.

**Fig. 2:**
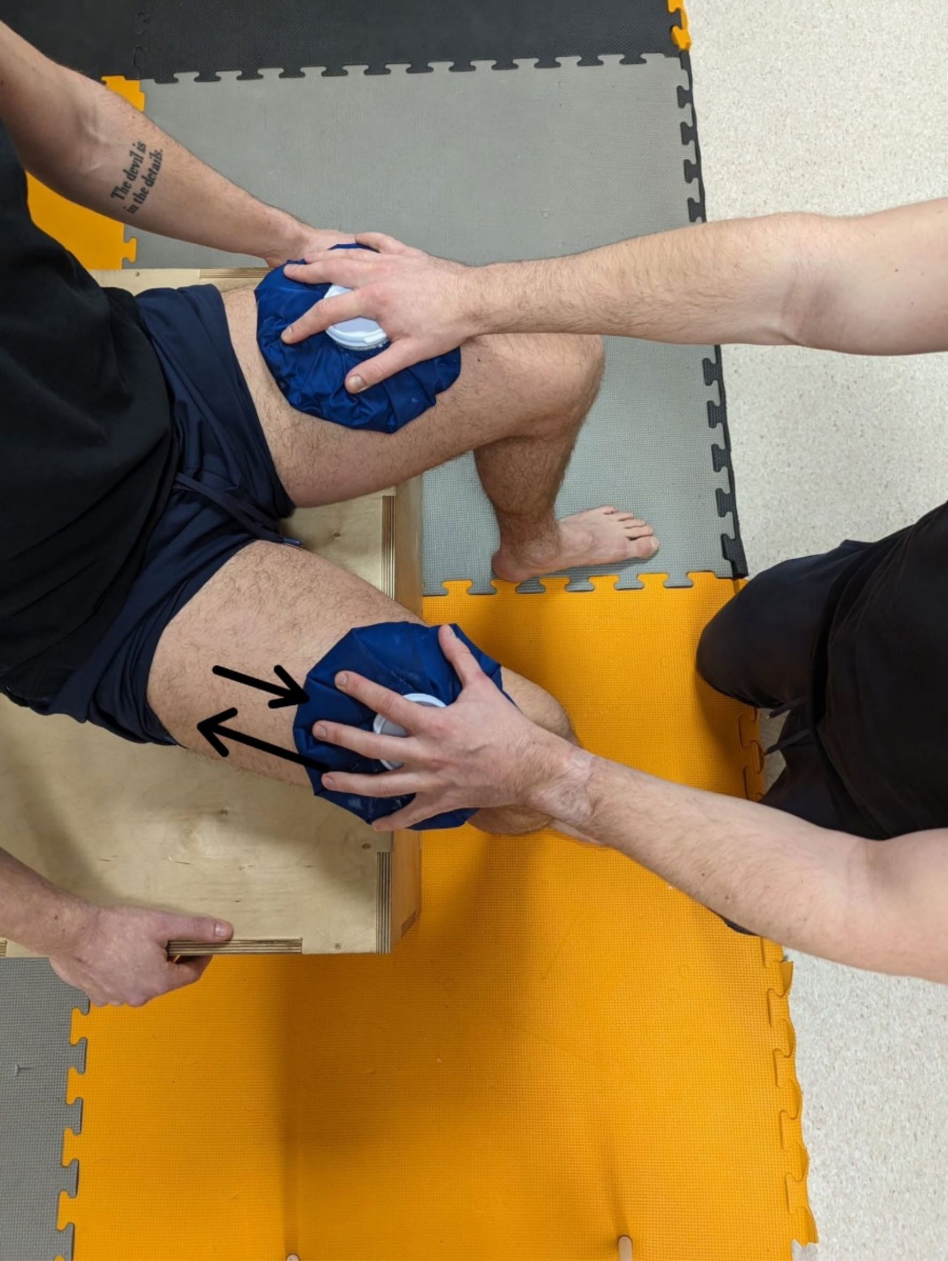
Ice massage intervention scheme.

**Fig. 3:**
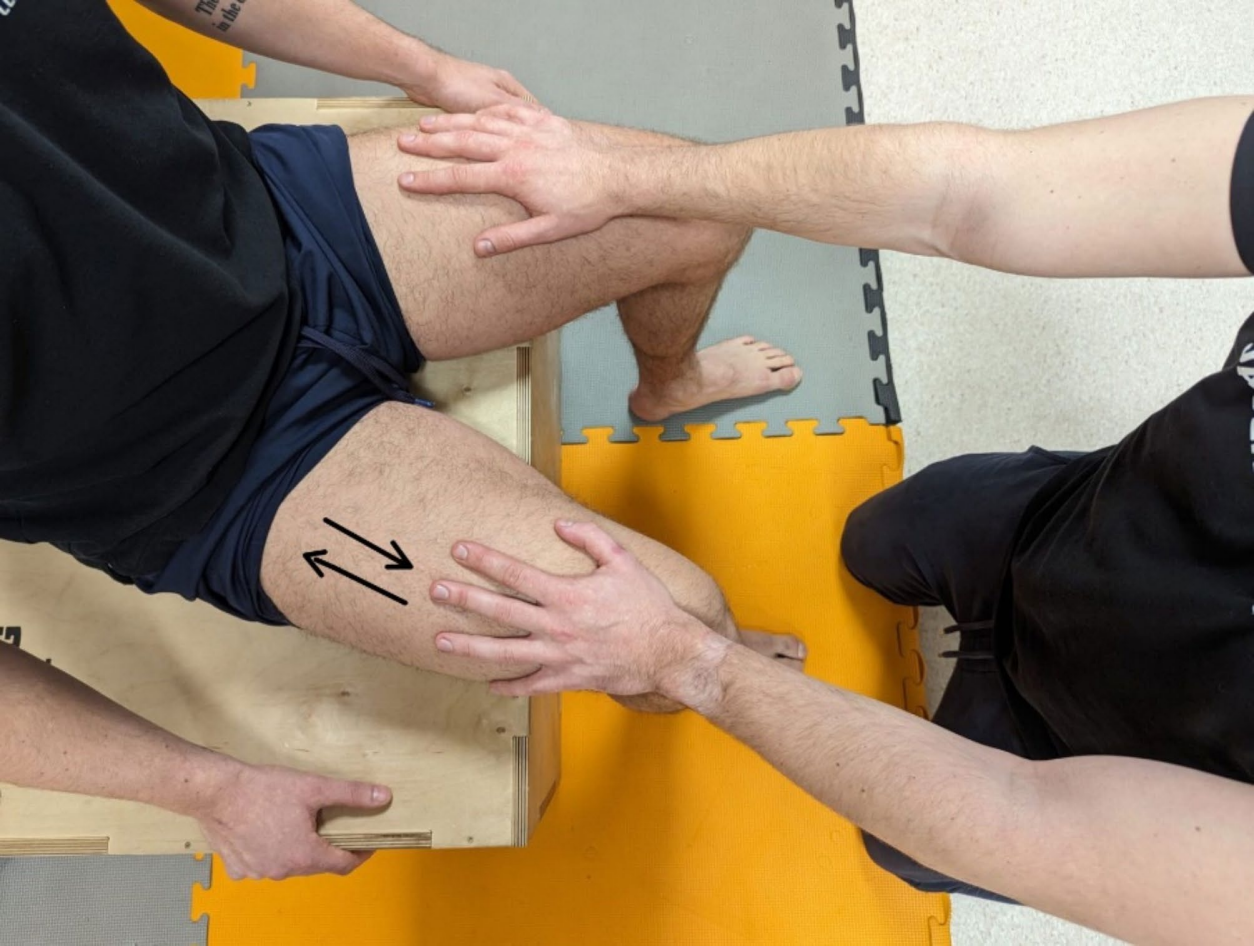
Massage intervention scheme.

Recovery intervention in each break between sets of exercises:

1. Ice massage session (Ice). During this session, one-minute compresses with ice bags were applied in each break between exercises. A back-and-forth rubbing motion was applied with moderate pressure (3 on a scale of 1–5) in one direction (distal-proximal), covering the entire muscle surface. (Fig. 2)
2. Dry massage session (Massage). During this session, a one-minute manual back-and-forth rubbing motion of the quadriceps muscles with moderate pressure (3 on a scale of 1–5) was applied, covering the entire surface of the muscle (Fig. 3).
3. Control session (Control): During each break between exercises, the athlete was sitting on a stool and took a one-minute passive rest.

The same professional physiotherapist performed the ice and dry massage interventions and was instructed to apply the same pressure and use the same technique for every intervention and participant. The interventions were carried out during the four breaks between the five sets of the fatigue protocol. The intervention protocol was developed based on familiar strategies used in breaks between combat sports efforts. The goal was to replicate the conditions of competition in the octagon as much as possible during an MMA championship fight^6^.

### Measurements

Accumuniq BC720 body composition analyzer (South Korea 2019) was used to assess the anthropometric characteristics of the study participants. Measurements were taken at one point in the medial part of both rectus femoris muscles under ultrasound guidance (USG-SONOSCAPE E2, China) to locate the broadest cross-section of the rectus femoris muscle (RF)^8^. This location was marked with a marker. Measurements were taken: (i) at rest – before the warm-up procedure; (ii) immediately after the last set of fatigue protocol (post-exercise). All measurements were taken in the same standardized supine position with extended hip and knee joints on a medical couch. The measurements were performed in the following order: Reactive Strength Index (RSI - m×s^− 1^), biomechanical properties: muscle tone (T - Hz), elasticity (E – arb - relative arbitrary unit), stiffness (S - N/m), pressure pain threshold, (PPT - N/cm²) and the number of jumps performed in the whole fatigue protocol was recorded (n).

### Reactive strength index (RSI - m×s^− 1^)

The measurement was performed using the VALD Force Decks ground reaction force plate (Vald-Performance, Australia, 2012), which is highly reliable and repeatable with a good or excellent intraclass correlation coefficient > 0.75)^47,48^. RSI describes an individual’s ability to quickly change muscle contraction from eccentric to concentric and is intended to assess the athlete’s reactive strength. Participants had to jump off a 50 cm box and perform a maximum vertical jump after landing on the force plate^8^. Before each RSI measurement, except for the one performed after the fatigue protocol, participants performed a 5-minute warm-up of 3 min of stationary cycling and leg stretching. A research assistant instructed all athletes on the jumping technique and performed three attempts before taking the measurements^49^. The best result was used in further analysis. The following formula was used to calculate RSI: RSI = Jump height/Contact time (m×s^− 1^)^54^.

### Biomechanical properties

A MyotonPRO (Myoton AS, Estonia, 2021) was used to assess the biomechanical properties of the rectus femoris muscle (T, S, and E). It is a digital palpation device consisting of a body and a depth probe (Ø 3 mm)^51^. MyotonPro uses the dynamic mechanical response method, which consists of a precise mechanical impulse, recording the dynamic response of the tissue in the form of a physical signal of displacement and acceleration of oscillations, and then calculating the parameters characterizing the studied biomechanical properties^52^. Scientific literature has confirmed its high reliability and repeatability (ICC reported from 0.52 to 0.99, mostly good or excellent)^7,53^. The probe is applied perpendicularly to the tissue, with an initial pressure of about (0.18 N), and the device releases a mechanical impulse (0.4 N, 15 ms), deforming the tissue for a short time. The algorithm calculates tissue vibrations, the amount of its deformation, the time to return to the resting position, and the damping of tissue vibrations, determining relative values ​​for specific parameters of muscle stiffness, tension, and elasticity^54^.

### Pressure pain threshold (PPT - N/cm^²^)

PPT was measured using the FPIX algometer (Wagner Instruments, Greenwich, CT, USA, 2013). Determining the pressure pain threshold is an attempt to objectively measure one of the parameters describing pain phenomena as a minimal force inducing pain^55^. This method was shown to have high reliability (ICC > 0.9)^56^. According to the manufacturer’s instructions, these measurements were performed on the rectus femoris (RF). Participants underwent a pressure probe test (*r* = 4 mm) three times, inducing compressive forces in a marked area that did not change during the study. The force value (N/cm^2^) was digitally displayed on the screen and calculated as the average of the three measurements. The pressure was applied until the test stimulus was signaled as unpleasant^8^.

### Number of jump repetitions

Assessing muscle fatigue through repetitions counts is a critical aspect of training protocols. Research indicates that prescribing a specific repetitions count can be helpful but has limitations^57^. Namely, it may not accurately reflect individual fatigue levels, which can vary significantly between individuals^58^. Therefore, in our assessment protocol, in addition to assessing fatigue through repetitions counts, we included RSI assessments to provide a more standardized approach. We evaluated the difference in jumps between the first and last sessions.

### Statistical analysis

Means and standard deviations were used to represent the average and the typical spread of values for all analyzed data. The normality of the data distribution was verified using the Shapiro-Wilk test. Homoscedasticity was tested using the Levene test. Compound symmetry, or sphericity, was checked using the Mauchly test. When the assumption of sphericity was not met, the significance of F-ratios was adjusted according to the Greenhouse–Geisser procedure. A two-way ANOVA with repeated measures: Group (Ice, Massage, Control) × Measure (Rest, Post-ex) was used to examine the changes in examined variables. Post-hoc tests with Bonferroni correction and ± 95% confidence intervals (CI) for absolute and percentage differences (Δ) were used to analyze the group and pairwise comparisons when a significant main effect or interaction was found. The significance level was set to *p* < 0.05 for all analyses. Effect sizes for ANOVA were calculated using partial eta squared ($\:{\eta\:}_{p}^{2}$) and interpreted according to the following criteria: if 0 ≤ $\:{\eta\:}_{p}^{2}$ < 0.05, there is no effect; if 0.05 ≤ $\:{\eta\:}_{p}^{2}$ < 0.26, the effect is minimal; if 0.26 ≤ $\:{\eta\:}_{p}^{2}$ < 0.64, the effect is moderate; and if $\:{\eta\:}_{p}^{2}$ ≥ 0.64, the effect is strong. Effect sizes for pairwise comparisons were calculated using Cohen’s d and interpreted as trivial (< 0.2), small (≥ 0.2), moderate (≥ 0.5), and large (≥ 0.8)^59^. A priori power analysis was conducted with the G*Power^60^. The repeated measure ANOVA within-between interactions with an effect size of at least 0.25, α = 0.05, and 1-β = 0.95 gave a statistical power of 95.3% and the total sample size of 66 subjects (counting by that matter, in the cross-over procedure there were 90 tested). In addition to traditional statistical analyses, responder analysis was conducted to identify individuals exhibiting clinically meaningful changes (minimal detectable change, MDC) in biomechanical parameters across different intervention groups. Furthermore, the relationships between variables were examined using Pearson’s linear correlation analysis. All the statistical analyses were conducted using RStudio (version 2024.04.2+764; RStudio Team, 2020)^61^ and R (version 4.4.1; R Core Team, 2024)^62^. We used a linear mixed-effects models (LMM) analysis, considering the within-subject and within-group differences. LMM were fit using the *afex* package (version 1.4-1)^63^ with p-values estimated using the *emmeans* package (version 1.10.4;^64^). All figures were created using the *ggplot2* package (Version 3.5.1)^65^.

## Results

At rest, no significant differences were observed between groups (Ice vs. Massage, Ice vs. Control, and Massage vs. Control) for the analyzed variables: RSI (*p* = 0.592, *p* = 0.562, and *p* = 0.999, respectively), T (*p* = 0.758, Ice vs. Massage), E (*p* = 0.498, *p* = 0.711, and *p* = 0.999, respectively), S (*p* = 0.215, *p* = 0.346, and *p* = 0.999, respectively), and PPT (*p* = 0.999, Ice vs. Control and *p* = 0.061, Massage vs. Control). Prior to exertion, there were also no significant differences in the number of jumps performed by participants across groups (*p* = 0.848, *p* = 0.999, and *p* = 0.999, respectively for Ice vs. Massage, Ice vs. Control, and Massage vs. Control). However, significant differences were noted in the resting values for T between the Ice vs. Control and Massage vs. Control groups (*p* < 0.001, and *p* < 0.001, respectively) as well as for PPT between the Ice vs. Massage groups (*p* = 0.035). Table 2 presents the descriptive and comparative characteristics for all variables examined.

**Table 2:** Comparison of results recorded at rest and post-exercise.

variable	group	rest	post-ex	*p*-value	Δ (± 95 CI )	Δ% (± 95 CI )	ES (± 95 CI )
RSI (m×s^− 1^)	I	2.37 ± 0.11	1.85 ± 0.09	< 0.0001	0.52 (0.49; 0.56)	28.3 (26.2; 30.4)	1.98 (1.43; 2.53)
	M	2.35 ± 0.11	2.03 ± 0.12	< 0.0001	0.32 (0.28; 0.36)	15.9 (13.6; 18.2)	1.21 (0.85; 1.57)
	C	2.35 ± 0.12	1.63 ± 0.10	< 0.0001	0.73 (0.67; 0.78)	45.1 (40.8; 49.4)	2.75 (1.98; 3.52)
T (Hz)	I	15.92 ± 1.28	17.69 ± 1.10	< 0.0001	− 1.77 (− 2.07; − 1.47)	− 10.0 (− 11.6; − 8.4)	− 0.66 (− 0.87; − 0.45)
	M	15.55 ± 1.22	16.33 ± 1.28	< 0.0001	− 0.78 (− 0.96; − 0.60)	− 4.7 (− 5.8; − 3.7)	− 0.29 (− 0.40; − 0.19)
	C	14.51 ± 0.82	18.61 ± 0.68	< 0.0001	− 4.09 (− 4.52; − 3.66)	− 21.9 (− 24.0; − 19.8)	− 1.54 (− 1.98; − 1.09)
E (arb)	I	274.77 ± 19.96	293.50 ± 20.13	< 0.0001	− 18.73 (− 21.43; − 16.03)	− 6.4 (− 7.3; − 5.5)	− 0.41 (− 0.54; − 0.29)
	M	273.57 ± 18.08	282.93 ± 17.26	< 0.0001	− 9.37 (− 10.52; − 8.22)	− 3.3 (− 3.8; − 2.9)	− 0.21 (− 0.27; − 0.15)
	C	269.67 ± 17.34	308.33 ± 17.96	< 0.0001	− 38.67 (− 42.91; − 34.42)	− 12.5 (− 13.8; − 11.2)	− 0.85 (− 1.10; − 0.61)
S (N×m^− 1^)	I	1.48 ± 0.25	1.66 ± 0.25	< 0.0001	− 0.18 (− 0.21; − 0.15)	− 10.9 (− 12.8; − 9.0)	− 0.29 (− 0.38; − 0.20)
	M	1.59 ± 0.24	1.68 ± 0.24	< 0.0001	− 0.09 (− 0.10; − 0.07)	− 5.3 (− 6.2; − 4.3)	− 0.14 (− 0.19; − 0.10)
	C	1.6 ± 0.29	1.96 ± 0.25	< 0.0001	− 0.36 (− 0.40; − 0.32)	− 18.8 (− 21.3; − 16.3)	− 0.58 (− 0.75; − 0.41)
PPT (N/cm²)	I	95.16 ± 3.85	82.94 ± 2.83	< 0.0001	12.22 (10.40; 14.04)	14.9 (12.6; 17.2)	1.38 (0.96; 1.81)
	M	91.85 ± 4.64	77.27 ± 3.56	< 0.0001	14.58 (12.55; 16.61)	19.1 (16.3; 21.9)	1.65 (1.15; 2.15)
	C	94.67 ± 3.23	64.18 ± 3.24	< 0.0001	30.49 (28.98; 32.00)	47.8 (44.8; 50.8)	3.45 (2.51; 4.40)
J (n)	I	27.47 ± 2.46	16.73 ± 0.98	< 0.0001	10.73 (9.83; 11.64)	64.5 (58.8; 70.2)	2.48 (1.78; 3.18)
	M	27.63 ± 2.40	17.23 ± 1.10	< 0.0001	10.40 (9.61; 11.19)	60.6 (55.8; 65.4)	2.40 (1.73; 3.08)
	C	27.33 ± 1.88	14.3 ± 1.09	< 0.0001	13.03 (12.18; 13.89)	92.4 (84.5; 100.2)	3.01 (2.18; 3.85)

RSI = Reactive Strength Index, T = tension, E = elasticity, S = stiffness, PPT = pressure pain threshold, J = number of jumps performed; I = Ice, M = Massage, C = Control, Δ = absolute difference, Δ% = percentage difference, CI = confidence intervals, ES = Cohen’s d effect size.

**Fig. 4:**
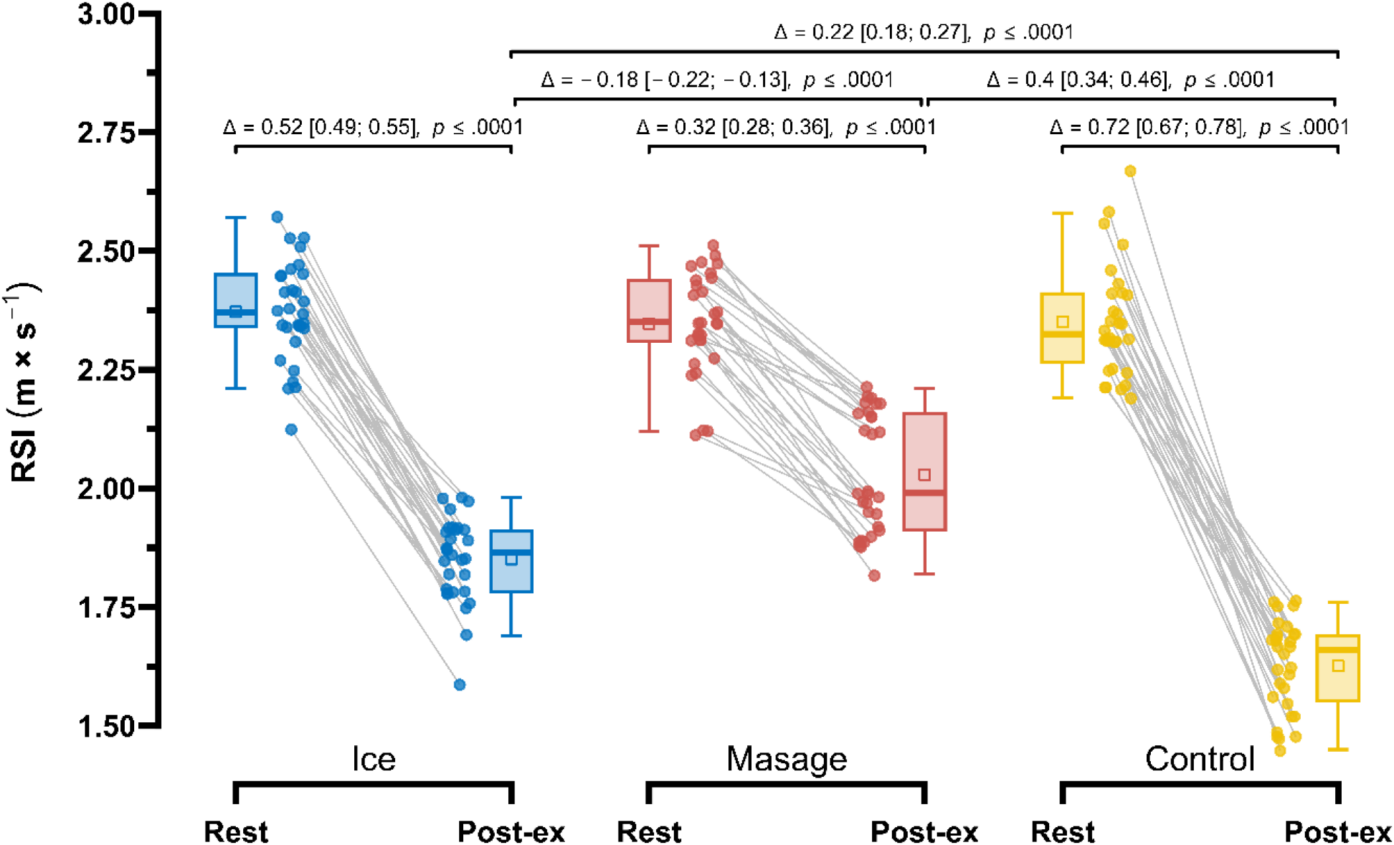
Comparison of changes in the Reactive Strength Index, RSI (m×s^− 1^) in the Control, Massage, and Ice groups at rest and post-exercise period.

Post-exercise, the smallest decrease in the Reactive Strength Index (RSI) (Fig. 4) was observed in the Massage group (2.35 ± 0.11 m×s^− 1^ vs. 2.03 ± 0.12 m×s^− 1^; Δ = 0.32 m×s^− 1^ (15.9%), *p* < 0.001), followed by the Ice group (2.37 ± 0.11 m×s^− 1^ vs. 1.85 ± 0.09 m×s^− 1^; Δ = 0.52 m×s^− 1^ (28.3%), *p* < 0.001). Repeated measures ANOVA revealed a significant main effect of Group (F_1.58, 45.89_ = 93.54; *p* < 0.001; $\:{\eta\:}_{p}^{2}$ = 0.76), Measurement (F_1.00, 29.00_ = 1041.35; *p* < 0.001; $\:{\eta\:}_{p}^{2}$ = 0.97), as well as their interaction (F_1.45, 42.18_ = 120.87; *p* < 0.001; $\:{\eta\:}_{p}^{2}$ = 0.81). In post-exercise measurements, RSI in the Massage group was higher compared to the Control group by 0.40 m×s^−1^ (25.1%), *p* < 0.001, ES = 1.53 (large), and also higher than in the Ice group by 0.18 m×s^− 1^ (8.6%), *p* < 0.001, ES = 0.68 (large). Additionally, in the Ice group, a significantly higher value of the Reactive Strength Index (RSI) was recorded post-exercise compared to the Control group by 0.22 m×s^− 1^ (14.1%), *p* < 0.0001, ES = 0.85 (large).

**Fig. 5:**
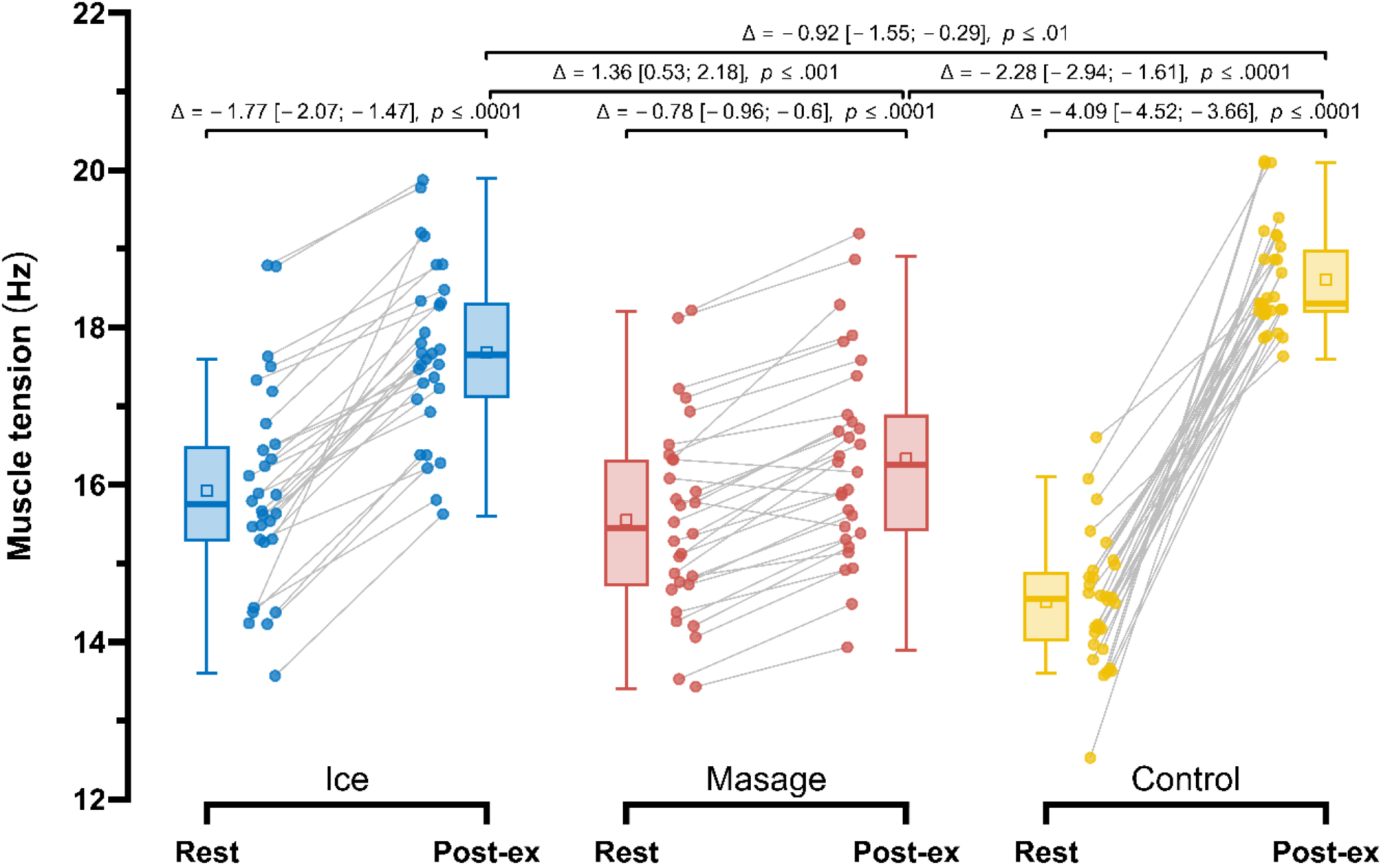
Comparison of changes in muscle tension, T (Hz) in the Control, Massage, and Ice groups at rest and post-exercise period.

Post-exercise, the smallest increase in muscle tension (T) (Fig. 5) was observed in the Massage group (15.55 ± 1.22 Hz vs. 16.33 ± 1.28 Hz; Δ = −0.78 Hz (− 4.7%), *p* < 0.001), followed by the Ice group (15.92 ± 1.28 Hz vs. 17.69 ± 1.1 Hz; Δ = −1.77 Hz (− 10.0%), *p* < 0.001). Repeated measures ANOVA revealed a significant main effect of Group (F_1.76, 50.96_ = 5.73; *p* < 0.01; $\:{\eta\:}_{p}^{2}$= 0.16), Measurement (F_1.00, 29.00_ = 565.48; *p* < 0.001; $\:{\eta\:}_{p}^{2}\:$= 0.95), as well as their interaction (F_1.78, 51.70_ = 122.40; *p* < 0.001; $\:{\eta\:}_{p}^{2}$ = 0.81). In post-exercise measurements, muscle tension in the Massage group was significantly lower compared to the Control group by 2.28 Hz (12.1%), *p* < 0.0001, ES = 0.85 (large), and also lower than in the Ice group by 1.36 Hz (9.0%), *p* < 0.001, ES = 0.51 (moderate). Additionally, in the Ice group, post-exercise muscle tension was significantly lower compared to the Control group by 0.92 Hz (4.8%), *p* < 0.01, ES = 0.35 (small).

**Fig. 6:**
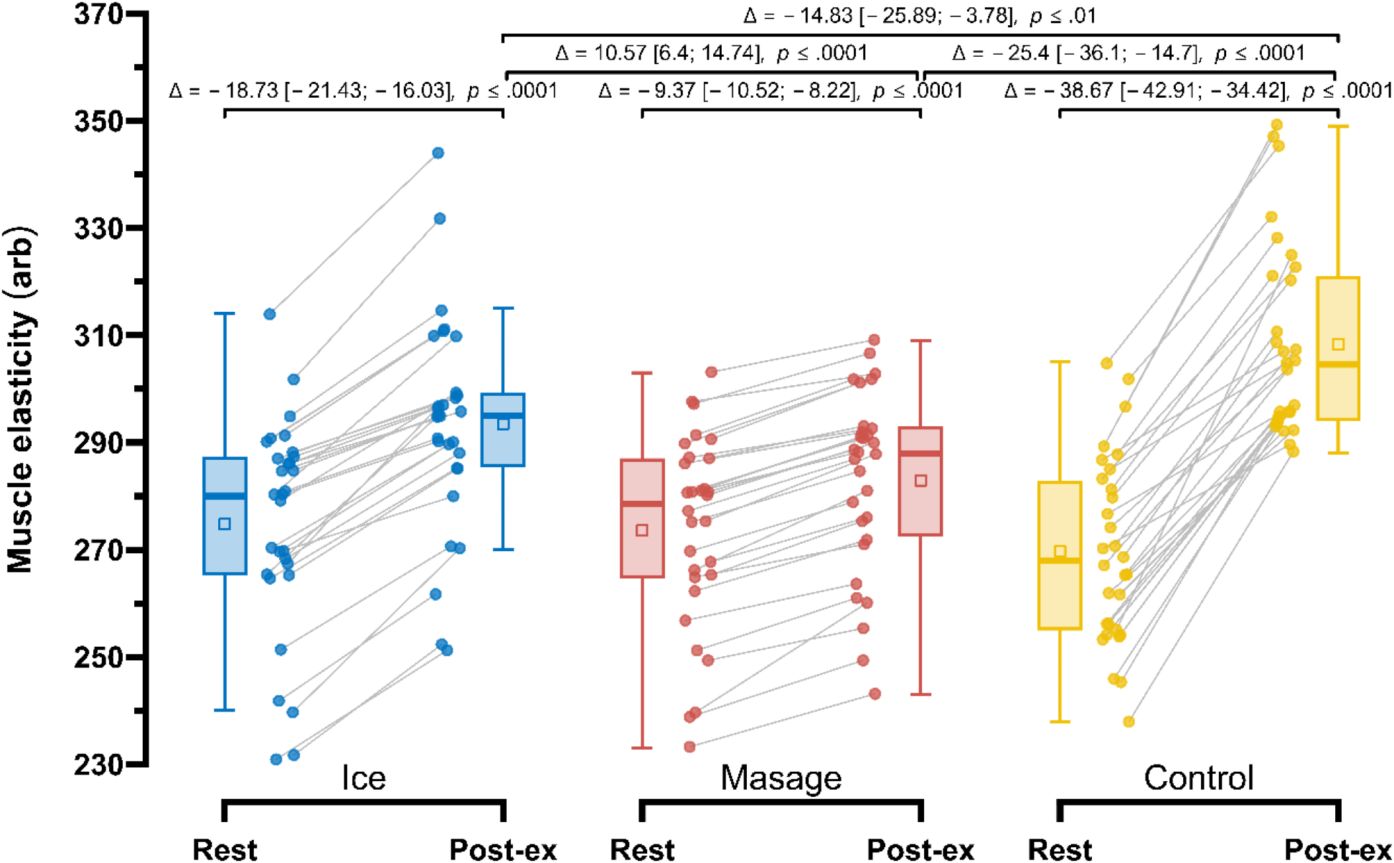
Comparison of changes in muscle elasticity, E (arb) in the Control, Massage, and Ice groups at rest and post-exercise.

Post-exercise, the smallest increase in muscle elasticity (E) (Fig. 6) was observed in the Massage group (273.57 ± 18.08 arb vs. 282.93 ± 17.26 arb; Δ = −9.37 arb (− 3.3%), *p* < 0.0001), followed by the Ice group (274.77 ± 19.96 arb vs. 293.5 ± 20.13 arb; Δ = −18.73 arb (− 6.4%), *p* < 0.0001). Repeated measures ANOVA revealed a significant main effect of Group (F_1.11, 32.12_ = 5.03; *p* < 0.05; $\:{\eta\:}_{p}^{2}$ = 0.15), Measurement (F_1.00, 29.00_ = 654.96; *p* < 0.001; $\:{\eta\:}_{p}^{2}$ = 0.96), as well as their interaction (F_1.60, 46.44_ = 109.33; *p* < 0.001; $\:{\eta\:}_{p}^{2}$ = 0.79). In post-exercise measurements, muscle elasticity in the Massage group was significantly lower compared to the Control group by 25.40 arb (8.0%), *p* < 0.0001, ES = 0.56 (moderate), and also lower than in the Ice group by 10.57 arb (3.7%), *p* < 0.0001, ES = 0.23 (small). Additionally, in the Ice group, post-exercise muscle elasticity was significantly lower compared to the Control group by 14.83 arb (4.6%), *p* < 0.01, ES = 0.33 (small).

**Fig. 7:**
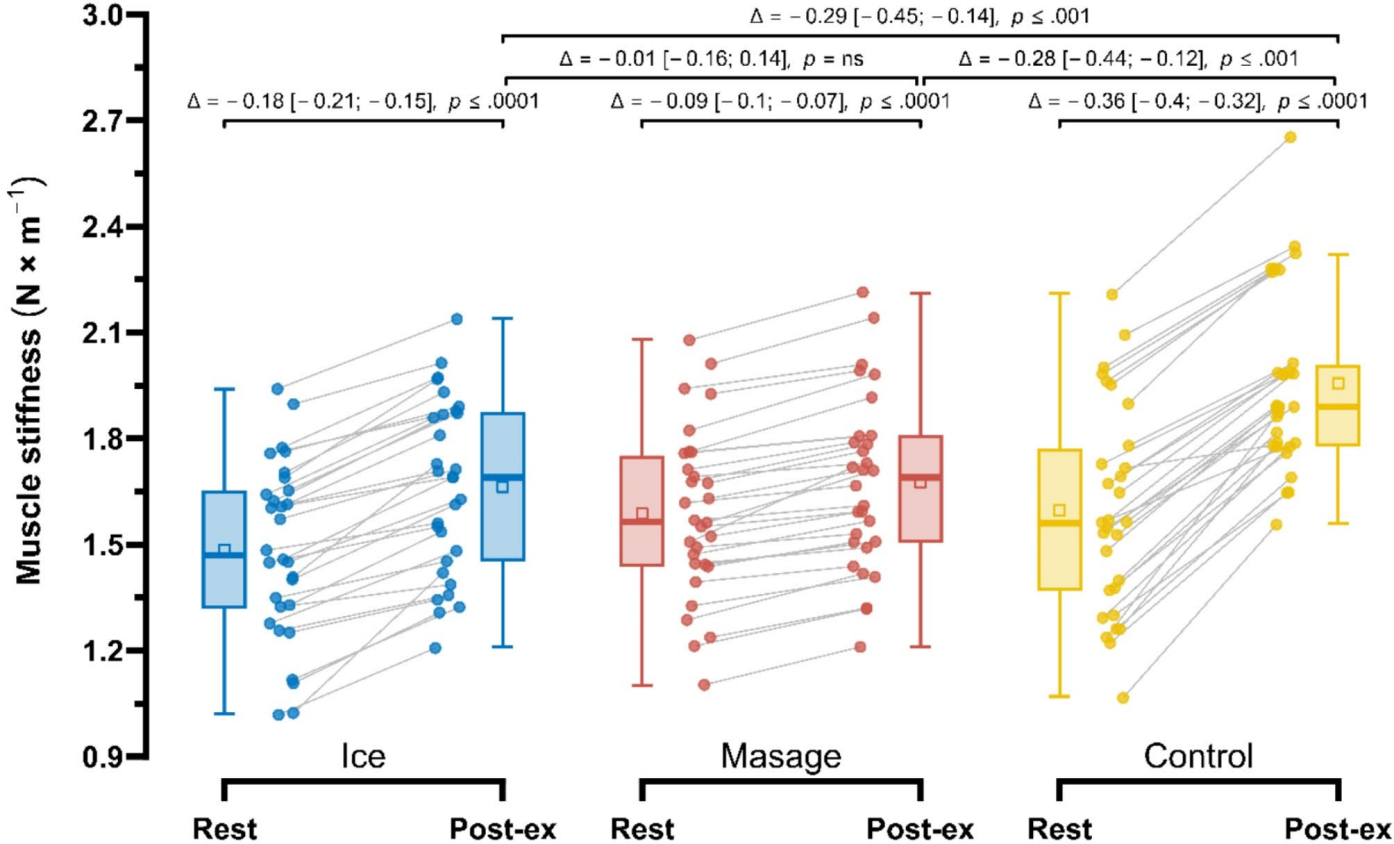
Comparison of changes in muscle stiffness, T (N×m^− 1^) in the Control, Massage, and Ice groups at rest and post-exercise.

Post-exercise, the smallest increase in muscle stiffness (S) (Fig. 7) was observed in the Massage group (1.59 ± 0.24 N×m^− 1^ vs. 1.68 ± 0.24 N×m^− 1^; Δ = −0.09 N×m^− 1^ (− 5.3%), *p* < 0.0001), followed by the Ice group (1.48 ± 0.25 N×m^− 1^ vs. 1.66 ± 0.25 N×m^− 1^; Δ = −0.18 N×m^− 1^ (− 10.9%), *p* < 0.0001). Repeated measures ANOVA revealed a significant main effect of Group (F_1.95, 56.48_ = 5.64; *p* < 0.01; $\:{\eta\:}_{p}^{2}$ = 0.16), Measurement (F_1.00, 29.00_ = 679.15; *p* < 0.001; $\:{\eta\:}_{p}^{2}$ = 0.96), as well as their interaction (F_1.53, 44.45_ = 85.23; *p* < 0.001; $\:{\eta\:}_{p}^{2}$ = 0.75). In post-exercise measurements, muscle stiffness in the Massage group was significantly lower compared to the Control group by 0.28 N×m^−1^ (13.1%), *p* < 0.001, ES = 0.45 (small), whereas the difference compared to the Ice group was not significant, amounting to 0.01 N×m^− 1^ (1.2%), *p* = 0.999, ES = 0.02 (trivial). Additionally, in the Ice group, post-exercise muscle stiffness was significantly lower compared to the Control group by 0.29 N×m^− 1^ (13.9%), *p* < 0.01, ES = 0.47 (small).

**Fig. 8:**
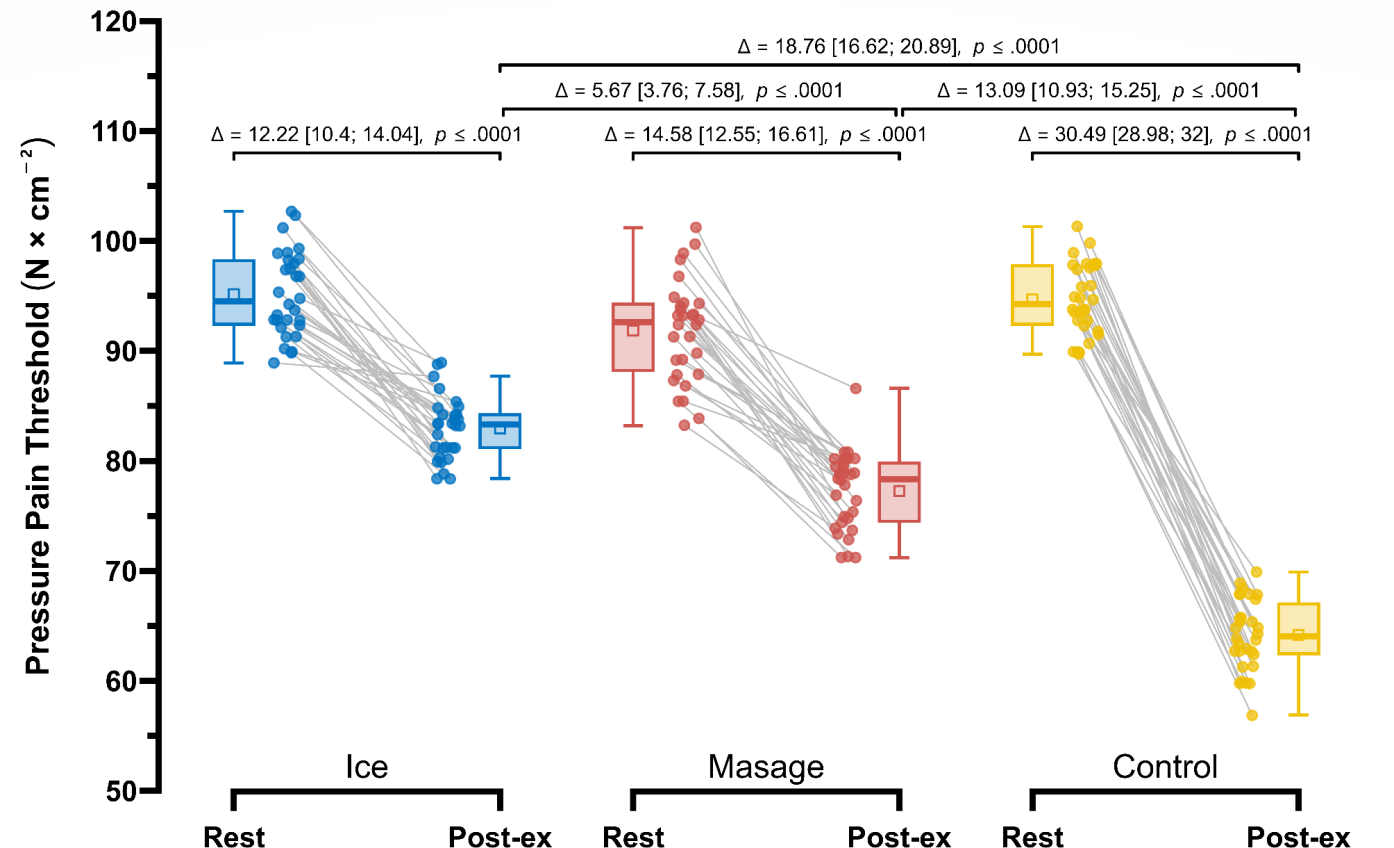
Comparison of pressure pain threshold, PPT (N×cm^-2^) in the Control, Massage, and Ice groups at rest and post-exercise.

Post-exercise, the smallest decrease in pressure pain threshold (PPT) (Fig. 8) was observed in the Ice group (95.16 ± 3.85 N/cm^2^ vs. 82.94 ± 2.83 N/cm^2^; Δ = 12.22 (N/cm^2^)(14.9%), *p* < 0.001), followed by the Massage group (91.85 ± 4.64 (N/cm^2^)vs. 77.27 ± 3.56 (N/cm^2^); Δ = 14.58 N/cm^2^ (19.1%), *p* < 0.001). Repeated measures ANOVA revealed a significant main effect of Group (F_1.78, 51.57_ = 90.93; *p* < 0.001; $\:{\eta\:}_{p}^{2}$ = 0.76), Measurement (F_1.00, 29.00_ = 1571.42; *p* < 0.001; $\:{\eta\:}_{p}^{2}$ = 0.98), as well as their interaction (F_1.95, 56.44_ = 121.44; *p* < 0.001; $\:{\eta\:}_{p}^{2}$ = 0.81). In post-exercise measurements, the pressure pain threshold (PPT) in the Ice group was significantly higher compared to the Control group by 18.76 (N /cm^2^)(29.6%), *p* < 0.001, ES = 2.12 (large), and also higher than in the Massage group by 5.67 (N/cm^2^)(7.53%), *p* < 0.001, ES = 0.64 (large). Additionally, in the Massage group, the post-exercise pressure pain threshold was significantly higher compared to the Control group by 13.09 N/cm^2^ (20.7%), *p* < 0.0001, ES = 1.48 (large).

**Fig. 9:**
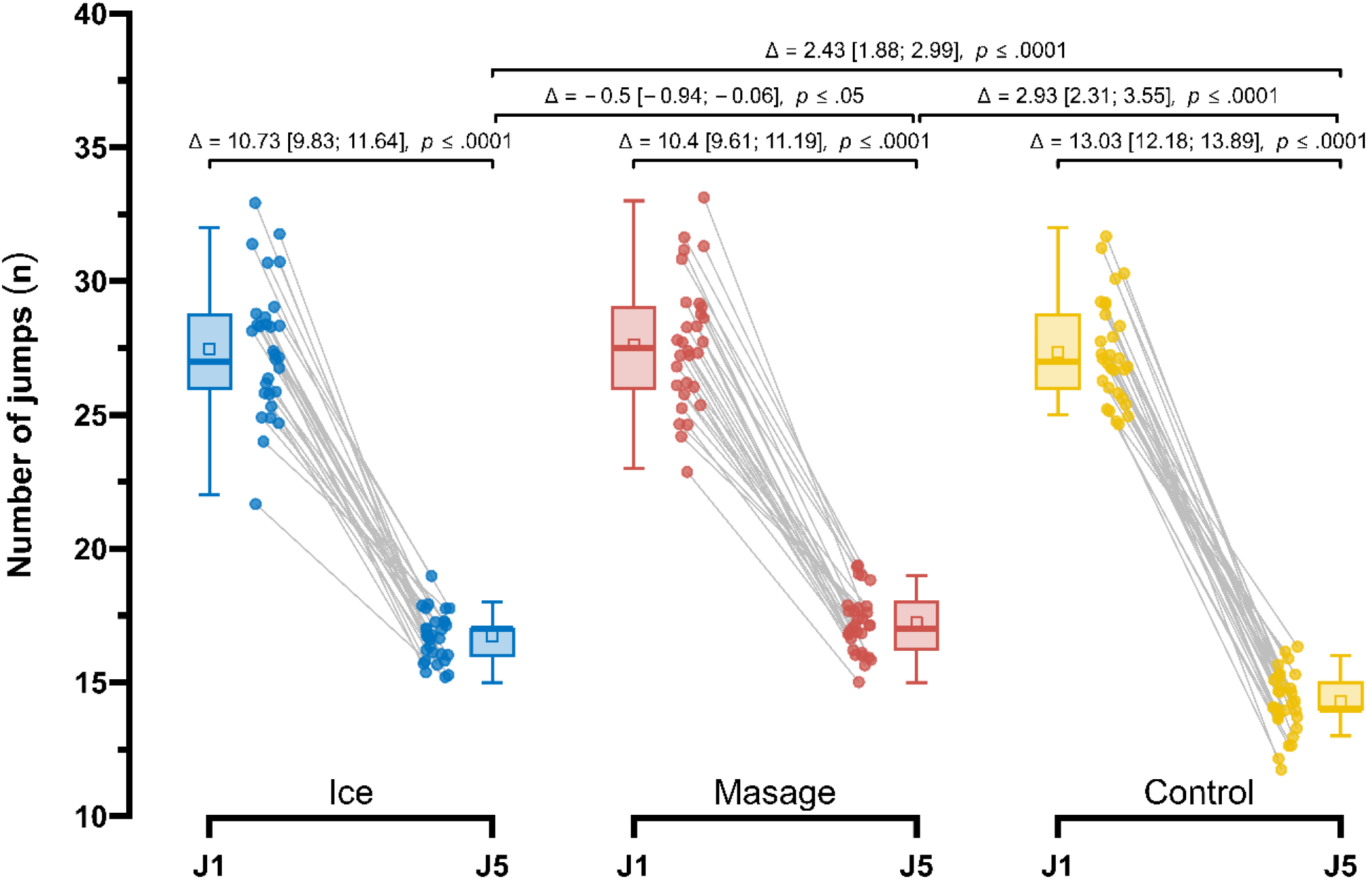
Comparison of changes in the number of jumps performed, J (n), in the Control, Massage, and Ice groups in the first and fifth series.

Post-exercise, the smallest decrease in the number of jumps performed (J) (Fig. 9) was observed in the Massage group (27.63 ± 2.4 vs. 17.23 ± 1.1; Δ = 10.40 (60.6%), *p* < 0.0001), followed by the Ice group (27.47 ± 2.46 vs. 16.73 ± 0.98; Δ = 10.73 (64.5%), *p* < 0.0001). In the Control group, the decrease in the number of jumps was the largest (27.33 ± 1.88 vs. 14.3 ± 1.09; Δ = 13.03 (92.4%), *p* < 0.0001). Repeated measures ANOVA revealed a significant main effect of Group (F*1.49*,* 43.19* = 55.65; *p* < 0.001; $\:{\eta\:}_{p}^{2}$ = 0.66), Measurement (F_1.00, 29.00_ = 997.48; *p* < 0.001; $\:{\eta\:}_{p}^{2}$ = 0.97), as well as their interaction (F_1.54, 44.78_ = 32.33; *p* < 0.001; $\:{\eta\:}_{p}^{2}$ = 0.53). In post-exercise measurements, the decrease of the number of jumps performed in the Massage group was significantly lower compared to the Control group by 2.93 (21.1%), *p* < 0.0001, ES = 0.68 (moderate), and also lower than in the Ice group by 0.50 (2.7%), *p* < 0.05, ES = 0.12 (trivial). Additionally, in the Ice group, the decrease in the number of jumps was significantly lower compared to the Control group by 2.43 (17.5%), *p* < 0.0001, ES = 0.56 (moderate).

**Fig. 10:**
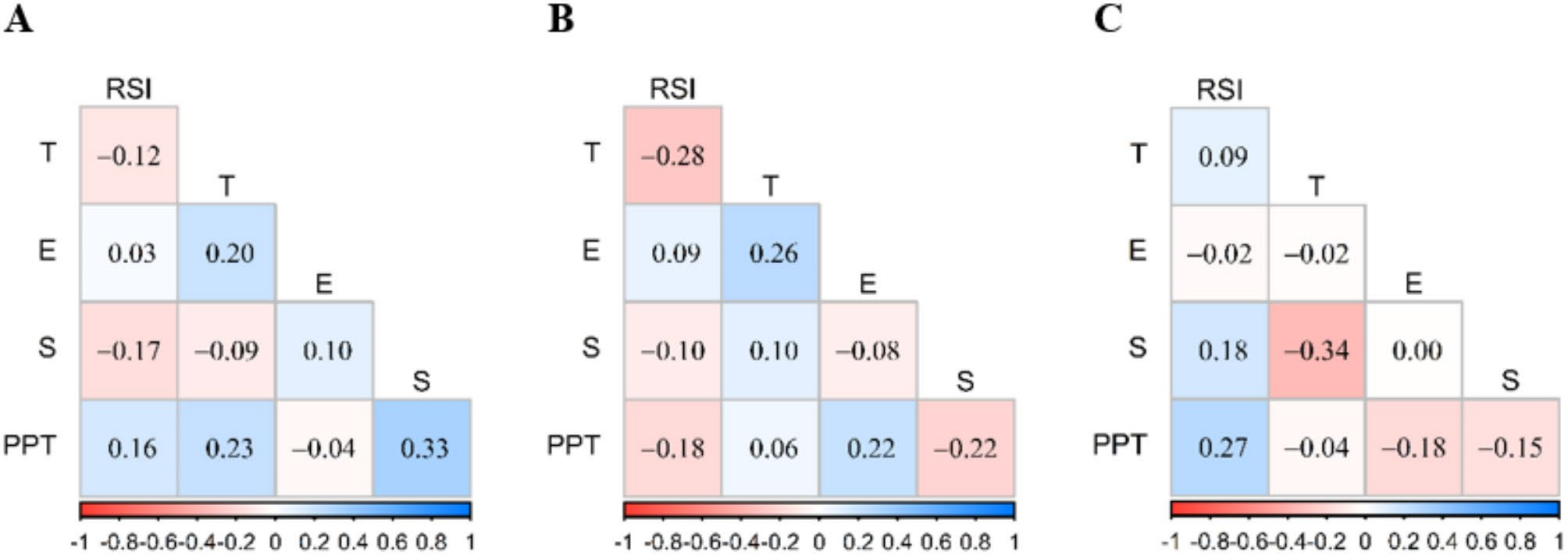
Pearson correlation matrix for biomechanical variables assessed after the intervention in the (A) ice group, (B) massage group, and (C) control group.

Figure 10 presents the linear correlation coefficients calculated for biomechanical variables measured after the intervention. None of the presented correlations reached statistical significance. Following the ice intervention (10 A), the highest correlation was observed between PPT and S (*r* = 0.33, *p* = 0.074). In contrast, after the massage intervention (10B), the highest correlation was found between RSI and T (*r* = − 0.28, *p* = 0.090). In the control group (10 C), the highest correlation was noted between S and T (*r* = − 0.34, *p* = 0.064).

Table 3 presents the results of the responder analysis for selected biomechanical variables and vertical jumps. It includes intraclass correlation coefficient (ICC) and MDC values, the number of responders in each group (Ice, Massage, Control), and the results of Pearson’s chi-square test assessing differences between groups. The highest number of responders was observed in RSI and Jump, where almost all participants exhibited a clinically significant change, regardless of the intervention applied. This indicates that the series of jumps induced a substantial decrease in reactive strength index (RSI) and power output in consecutive jumps (Jump). The lack of differences between groups suggests that neither ice cooling nor massage had a significant impact on recovery in these parameters. For T, significant differences were observed between groups (*p* < 0.001). In the Massage group, only 18 participants exceeded the MDC threshold, while in the Ice and Control groups, it was 30 participants. This suggests that the jump series led to a considerable increase in muscle stiffness, but massage may have mitigated this effect. The most pronounced differences were found for E. In the Control group, 26 participants exhibited a significant decrease in elasticity, while in the Ice group, only 6 participants were responders, and in the Massage group, there were 0 responders. This indicates that while the jumps themselves reduced muscle elasticity, Ice and Massage interventions helped prevent this decline, with massage completely counteracting the effect. The S parameter also showed substantial differences between groups (*p* < 0.001). In the Control group, 29 participants experienced increased muscle tension, whereas in the Massage group, only 12 participants showed this response, suggesting that massage effectively reduced post-exercise muscle tension, while ice cooling had a weaker effect (28 responders). For PPT, the number of responders was high and similar across all groups (29–30 participants), and the differences were not statistically significant (*p* = 0.364). This indicates that the jump series decreased pain tolerance regardless of the applied intervention. A similar trend was observed for Jump, where all participants (30 in each group) showed a significant response post-exercise, confirming that the series of jumps significantly impaired explosive strength, and the interventions were insufficient to counteract this decline (*p* = 1.000).

**Table 3:** Analysis of responders for Biomechanical variables and jumps.

Variables	ICC_(3,1)_ | MDC	Responders in group			Pearson’s $\:{\chi\:}^{2}$	*p*-value
		Ice	Masage	Control		
RSI	0.77 | 0.156	30	28	30	4.09	0.129
T	0.88 | 0.792	30	18	30	27.69	< 0.001
E	0.75 | 24.03	6	0	26	53.92	< 0.001
S	0.99 | 0.081	28	12	29	33.913	< 0.001
PPT	0.89 | 2.971	29	30	30	2.02	0.364
J	0.93 | 2.184	30	30	30	0.00	1.000

RSI = Reactive Strength Index, T = tension, E = elasticity, S = stiffness, PPT = pressure pain threshold, J = number of jumps performed; ICC – intraclass correlation coefficient, MDC = minimal detectable change.

## Discussion

Our study confirmed the effectiveness of the both tested regeneration methods in the immediate recovery of strength, change of biomechanical properties of muscles (stiffness, elasticity, muscle tone), and reduction of muscle pain, indicating slightly better results in the dry massage group. In case of reduction of muscle pain measured by PPT ice massage brought the most beneficial changes compared to the dry massage and control group.

In our study, both experimental groups (massage and ice) were subjected to eccentric fatigue of the quadriceps femoris muscles. Massage showed statistically significant, immediate differences comparing to the control group in reducing the level of fatigue measured by the RSI index and the number of jumps performed in series. In our case, dry massage showed the most excellent effectiveness. We observed positive effects on muscle biomechanical properties such as tension, stiffness, and elasticity between experimental interventions and controls, however Ice massage method brought better results in case of PPT. Muscle biomechanical properties are essential to muscle functions^66^. Although there are various hypotheses about the effect of exercise on short-term changes in muscle stiffness and elasticity, several in vivo studies demonstrate increased musculoskeletal system stiffness after eccentric exercise^67^. Although the mechanisms behind the biomechanical changes in muscle during exercise are not fully understood, changes at the bundle and single fiber level may be both a physiological adaptation and a possible protective mechanism^68^. Although the precise interaction between different muscles and bundles and fibers is not fully understood, it is likely that the adaptation is based on a combination of neurogenic and mechanical factors (including changes in collagen architecture and hydration)^69^.

Muscle recovery strategies can be used at different times: before, during, or after exercise^70^. Improving performance or reducing the rate of fatigue during high-intensity efforts in successive sets of repetitions is particularly important in combat sports^18^. First, athletes compete in 3 or 5 rounds with a one-minute break between them. During this time, coaches and physiotherapists often use cooling or massage of the most tired muscle groups. Second, athletes usually compete in tournaments and have to compete in several fights^5^. Therefore, determining which techniques are more effective and how they affect biomechanical properties is still debatable.

Integrating colling and dry massage techniques into recovery protocols serves two purposes: relieving muscle soreness and improving performance outcomes after exercise. These techniques utilize the physiological mechanisms of cold therapy and mechanotransduction^37,71^, which can induce microvascular responses^23^ thereby reducing inflammation^12,72^ and muscle pain immediately following intense physical activity^73^. Research indicates that although recovery protocols vary, the effectiveness of cooling methods may vary depending on individual factors such as genetics and fitness level^27^. Therefore, our study aimed to evaluate the immediate effects of these two most popular forms of muscle stimulation, often used in between-exercise intervals and between rounds of a sports fight.

Previous studies have shown conflicting results^12,74–76^. Esteves et al. found that no cooling strategy effectively improved exercise performance during high-intensity resistance exercise^70^. Exercise performance was positively modulated only when ice was applied to the neck. Their results may be a consequence of too short time of exposure to cooling^70^. Our results contradict this hypothesis. The methodology of cooling may be essential. We performed intensive ice rubbing in our study, whereas Esteves’s study used a static method.

Douzi et al. presented a meta-analysis showing that per-cooling improves ‘aerobic’ and ‘anaerobic’ exercise performance, with a significant benefit for ‘aerobic’ exercise^74^. The effect’s magnitude depends on the cooling application’s type and size. Most laboratory studies demonstrate significant improvements in exercise performance following pre-cooling or during exercise in increased humidity and high external temperature^77^. Although the dissimilar mechanisms of the effect of local cooling on fatigued muscles remain unclear, the prevailing consensus is that the application of cold during exercise reduces heat stress in muscle tissue by causing a temporary constriction of blood vessels and then their secondary dilation, which helps reduce local inflammation induced by excessive physical exertion^23,78–80^.

Although evidence that massage between exercises can directly increase motor skills and strength is limited^29,81,82^, it may influence recovery and psychological factors that indirectly support performance^17,21,28,83^. Although massage as a regeneration strategy is one of the most common and effective methods^83,84^, there are still ambiguities and gaps, especially in the reproducibility and repeatability of the method and the assessment of potential physiological mechanisms affecting tired muscles^85^. One is mechatronics, which converts mechanical stimuli into biochemical signals that promote healing and regeneration. This process is crucial to understanding how massage therapy can affect cell behavior and tissue repair, using the body’s natural responses to mechanical forces^33,86^.

The investigated recovery methods influence the efficiency and the effectiveness of muscle-to-tendon physical transduction^87^, joint range of motion^88^, and performance^42^ as well as the risk of injury^89^. For this reason, studies evaluating the effect of recovery methods to compensate for these changes are clinically relevant. Previous research has suggested that a musculoskeletal system with more excellent elasticity (less stiffness) has a greater capacity to lengthen, allowing it to absorb external forces and exert a moderating effect on energy production during movement^20,90^. The effects of massage on muscle stiffness reveal both immediate benefits and limitations in duration. Studies indicate that massage can lead to a short-term reduction in muscle stiffness, especially after exercise, but this effect is usually transient and may not last over time^91^. Mechanical pressure can help blood circulation by removing metabolic products caused by oxidative stress, thus reflexively activating an increase in muscle tone^92^. This is evident in the action of the parasympathetic system (assessed by heart rate, blood, and cardiac output) and the action of measured neurohormones and subjective questionnaires of the well-being of one’s own body^81^.

The physiological mechanisms underlying muscle pain during and after exercise are complex and multifaceted, involving various biochemical and cellular processes^93^. During intense muscle exercise, exceedingly eccentric exercise with previously unique movement pattern, micro-injuries of muscle fibers occur, leading to an inflammatory response. This response is characterized by releasing various inflammatory mediators, which cause swelling, muscle pain, and soreness, known as delayed onset muscle soreness (DOMS)^27,42^. Muscle tissue dysfunction leads to increased levels of creatine kinase (CK) in the blood, a biomarker of muscle damage. Increased CK levels correlate with the intensity of muscle injury and perceived pain^17,20^. Scientific literature describes many factors that have the impact on post-exercise muscle pain, including cooling and massage^29,94,95^.

Following acute exercise, skeletal muscle undergoes a variety of cellular and molecular responses that mediate adaptations and recovery, significantly influencing the sensation of muscle pain. One key aspect is the activation of signaling pathways associated with inflammation and muscle repair, which can exacerbate the sensation of pain and discomfort. As noted in the existing literature, skeletal muscle plasticity demonstrates a critical role of contractile loading in determining these responses, including the secretion of proinflammatory cytokines^28^. Our results show that ice massage is more effective in reducing muscle pain measured by PPT, and it can bring further effects in reduction of inflammatory processes.

Although commonly used, post-exercise recovery methods such as cooling or massage may hinder long-term adaptations by impairing anabolic signaling pathways necessary for muscle recovery and hypertrophy^8,17^. Some studies indicate that cold stimuli may inhibit muscle protein synthesis, suggesting that although they may provide short-term pain relief, they may impair overall recovery and adaptation processes^12,96^. The study of post-exercise pain mechanisms in humans is fraught with difficulties, and only limited insight can be achieved, especially regarding the role of central and peripheral sensitization^97^. However, assessment of reducing the subjective sensation of muscle pain by using an algesimeter, as was done in our case, and simultaneous assessment of biomechanical changes may become a good tool for in vivo analysis^98,99^.

The study of the effects and physiological mechanisms that govern inter-exercise muscle recovery using ice and dry massage underscores the complexity of muscle recovery processes in MMA athletes. Effective recovery protocols may be excellent and straightforward strategies for relieving eccentric exercise-induced muscle soreness and improving overall performance. While various studies indicate the equivocal nature of their effectiveness and the need for further research, our study indicates the practical utility of both forms of inter-exercise relief of muscle fatigue symptoms, with dry massage appearing slightly more effective than ice massage. Interestingly, the study indicates that passive recovery methods may not benefit significantly from overactive techniques, which challenge traditional practices. Ultimately, the results emphasize the need for a tailored approach to recovery that considers the individual athlete’s response and the intricacies of each recovery method. As evidence evolves, refining recovery strategies will be crucial to optimizing athletic performance and minimizing the risk of injury. The question if combining the dry massage and ice massage in short rest of 1 min between the rounds of an MMA fight will bring better results than solo dry massage or solo ice massage remains open. Although scientific knowledge about regeneration methods and their effectiveness is increasing, further research is undoubtedly needed to create evidence-based regeneration protocols for various sports disciplines.

## Practical application

Both dry massage and ice massage are recommended methods for improving recovery during rest periods in MMA and other combat sport. Both these methods are low cost and easy to use, but they differ in case of their effects according to PPT. Considering that – dry massage is recommended in case of excessive fatigue, and ice massage is recommended in case of increased pain. Not using any of the methods of supporting regeneration during breaks in an MMA fight is absolutely not recommended, because the fight causes significant fatigue, negatively affecting the muscles, and the use of any of the analyzed methods helps to effectively and easily eliminate these unfavorable changes.

## Limitations and future research directions

Although our study provides evidence to support ice and dry massage use in inter-exercise muscle recovery after eccentric fatigue compared to passive rest, it has limitations and future research directions were formulated. First of all, our study focused on a selected group of athletes. Therefore, it is necessary to conduct a future project using other sports disciplines and healthy and inactive individuals. Additionally, it is worth considering increasing the size of the groups and increasing age and gender diversity. Future studies should include additional biochemical measurements (e.g., creatine kinase) and other exercise protocols that do not require the advanced skills of performing plyometric training as in our study (e.g., cycle ergometry tests). The long-term implications of these recovery strategies in terms of injury prevention and athlete longevity should be examined in the dedicated longitudinal study. Research leading to the discovery of the exact mechanisms of the effects of heat and cold on human muscles should help determine why dry massage had a better effect on muscle biomechanical parameters in this case compared to ice massage. Ultimately, these implications emphasize the need for further research and a holistic approach to exercise programming that prioritizes physiological and psychological recovery components.

## Conclusions

Both ice massage and dry massage performed during 1 min brakes between 5 sets of box jumps eccentric exercises up till exhaustion significantly improves regeneration measured as muscle biomechanical parameters (stiffness, elasticity and muscle tone), preserve the lowering of the PPT, helps preserve the muscles ability to generate power in dynamic eccentric motion and help reduce muscle fatigue measured by the number of jumps performed till exhaustion in box jumps exercise when comparing to a passive break. The dry massage was more effective in case of biomechanical parameters compared to ice massage, and the ice massage was better in preventing the decrease of PPT compared to the dry massage. Both strategies are proven to be effective in highly trained MMA athletes and are recommended to use in rest brakes during real fight conditions.

## Data Availability

The datasets generated and/or analysed during the current study are available in the OSF data repository, https://osf.io/ukjgz? view_only=6d2e29d5de984ea295deef6068e8290d.
