# Author Correction: Immediate effect of ice and dry massage during rest breaks on recovery in MMA fighters: a randomized crossover clinical trial study

**DOI:** 10.1038/s41598-025-99707-0

**Published:** 2025-04-25

**Authors:** Robert Trybulski, Arkadiusz Stanula, Andriy Vovkanych, Jarosław Muracki, Hsing-Kuo Wang, Adrian Kużdżał

**Affiliations:** 1 Medical Department, Wojciech Korfanty Upper Silesian Academy, 40-659 Katowice, Poland; 2 Department of Physical Therapy and Ergotherapy, Ivan Boberkyj Lviv State University of Physical Culture, Lviv, 79007 Ukraine; 3 Laboratory of Sport Performance Analysis, Institute of Sport Sciences, Jerzy Kukuczka Academy of Physical Education in Katowice, 40-065 Katowice, Poland; 4 Institute of Physical Culture Sciences, Department of Physical Culture and Health, University of Szczecin, 70-453 Szczecin, Poland; 5 Provita Medical Center, 44-240 Żory, Poland; 6 School and Graduate Institute of Physical Therapy, National Taiwan University, Taipei, Taiwan; 7 Center of Physical Therapy, National Taiwan University, Taipei, Taiwan; 8 Institute of Health Sciences, College of Medical Sciences, University of Rzeszów, 35-310 Rzeszów, Poland

Correction to: *Scientific Reports* 10.1038/s41598-025-97194-x, published online 10 April 2025

The original version of this Article contained errors. The spelling of the author Robert Trybulski was incorrectly given as Robert Trybulsk.

Additionally, this Article contained a repeated error in the pressure pain threshold unit,

“(N/cm)”

now reads:

“N/cm^2^”

Finally, the article contained an error in the Figure 8 legend,

“Comparison of pressure pain threshold, PPT (N×cm) in the Control, Massage, and Ice groups at rest and post-exercise.”

now reads:

“Comparison of pressure pain threshold, PPT (N×cm^−2^) in the Control, Massage, and Ice groups at rest and post-exercise.”

The original Article has been corrected.

